# A Network-Based Data Integration Approach to Support Drug Repurposing and Multi-Target Therapies in Triple Negative Breast Cancer

**DOI:** 10.1371/journal.pone.0162407

**Published:** 2016-09-15

**Authors:** Francesca Vitali, Laurie D. Cohen, Andrea Demartini, Angela Amato, Vincenzo Eterno, Alberto Zambelli, Riccardo Bellazzi

**Affiliations:** 1 Dipartimento di Ingegneria Industriale e dell'Informazione, Università di Pavia, Pavia, Italy; 2 IRCCS-Fondazione S. Maugeri, Pavia, Italy; 3 Oncologia Medica, ASST Papa Giovanni XXIII, Bergamo, Italy; University of Texas at San Antonio, UNITED STATES

## Abstract

The integration of data and knowledge from heterogeneous sources can be a key success factor in drug design, drug repurposing and multi-target therapies. In this context, biological networks provide a useful instrument to highlight the relationships and to model the phenomena underlying therapeutic action in cancer. In our work, we applied network-based modeling within a novel bioinformatics pipeline to identify promising multi-target drugs. Given a certain tumor type/subtype, we derive a disease-specific Protein-Protein Interaction (PPI) network by combining different data-bases and knowledge repositories. Next, the application of suitable graph-based algorithms allows selecting a set of potentially interesting combinations of drug targets. A list of drug candidates is then extracted by applying a recent data fusion approach based on matrix tri-factorization. Available knowledge about selected drugs mechanisms of action is finally exploited to identify the most promising candidates for planning *in vitro* studies. We applied this approach to the case of Triple Negative Breast Cancer (TNBC), a subtype of breast cancer whose biology is poorly understood and that lacks of specific molecular targets. Our “in-silico” findings have been confirmed by a number of *in vitro* experiments, whose results demonstrated the ability of the method to select candidates for drug repurposing.

## Introduction

Over the past decades, advances in biological science have led to the generation of a large amount of molecular data at the level of genome, transcriptome, proteome, and metabolome, with the potential for greatly advancing patient care and clinical research, in particular concerning cancer. The characterization of thousands of disease cases has revealed that the majority of cancers harbors a cocktail of mutated or altered genes that work in concert to specify molecular pathways that lead to their genesis, maintenance, and progression [1]. Therefore, the identification of genes and proteins is not sufficient to fully understand the disease complexity, since it provides only a catalog of individual molecular components [2]. On the contrary, it is crucial to know how the individual components interact with each other, or how changes in external and internal conditions may dynamically alter the resulting complex behaviors.

In this context, system biology and bioinformatics can offer a suitable way of approaching the study of the disease, and, more ambitiously, the discovery of novel therapies by developing models that consider the whole pathophysiological picture without losing the key molecular details. Substantial advances have been achieved by integrating computational modeling with quantitative experimental data and knowledge with different methodological approaches, coming from statistics, machine learning and systems theory, particularly in the field of cancer system biology [3].

In recent years, methods based on a network description and analysis have shown to be able to provide an interesting strategy for drug design and repurposing [4–9]. Through a network-based approach, a complex system can be represented as a graph, where nodes correspond to the molecular entities of interest (e.g. proteins, drugs), while edges represent their interactions (e.g. physical interactions). Latest studies in network biology showed that systems underlying complex diseases are controlled by several biological concurrent processes and are robust against perturbations. Therefore, gene and protein networks seem ideal instruments for studying the repurposing of approved drugs, especially when jointly taking the wired nature of targeted biological systems [10].

As a consequence, network modeling can be also seen as a “natural” instrument to deal with the combination of drug repurposing and multi-target drug design. Multi-target drugs may be able to comprehensively target the pathological network of a disease and to amplify the final therapeutic success due to their treatment effects by synergy [11]. In fact, combinations of drugs with synergistic mechanisms of action should minimize drug resistance and maximize cellular effects [12]. Therefore, a bioinformatics, network-based, pipeline may have a crucial role to reduce the space of drug candidates and to select new potential disease therapies for complex diseases.

Recent approaches have demonstrated that many proteins are already targeted by more than one drug, suggesting that multi-target candidates can be automatically retrieved by analyzing the interactions among proteins, drugs, and diseases [8,13,14]. Other interesting methods have been developed to prioritize the most effective combinations of drugs and targets for experimental validation *in vivo* or *in vitro*. Such computational models represent a crucial enabling factor, since testing all possible drug combinations is unfeasible because their number increases exponentially with the drugs to be tested. It should be noted that the molecular response profiles, such as gene expression data, are currently still scarce for drug combinations, even if some efforts towards their prediction have been made [4]. In order to improve the integration of knowledge sources about drug behaviors, Huang et al. have proposed an evaluation tool, called *DrugComboRanker* [15]. In this study, the authors developed a synergistic score for drug combinations on the basis of the topological relatedness of drug targets in signaling networks, semantic similarity of gene ontologies and the dissimilarity of gene expression profiles of different drugs. The method was assessed on lung adenocarcinoma and HER2-subtype breast cancer and most of the top multi-target combinations were confirmed by literature reports [15].

Other approaches focus on the integration of drug-target network with the human disease network to reveal drug targets that are often involved in multiple diseases. These approaches can be useful to automatically repurpose drug and targets, too [16–18].

Network construction and analysis can also provide a computational framework for performing perturbation experiments *in silico* and to assess the global effect of targeted interventions. These experiments are aimed to predict the robustness of a network to perturbations that simulate pharmacological administrations.

A key disadvantage of drug discovery approaches based on the construction of protein, drug and disease networks, such the ones previously presented, is that they provide a static view of the problem. The cellular network underlying a specific disease is highly dynamic: in fact, molecules, proteins and gene associate, dissociate and interact. Taking into account these dynamics across pathways and networks is a very challenging problem, involving detailed experimental data and computational requirements [9]. In this context, pathways representing specific parallel, cross-talk or feedback of molecular structures are powerful elements to explore in depth the mechanisms underlying the drugs actions: these ones can be perceived as network perturbations whose effect should lead the pathophysiological networks back to their normal state. Thus, studies addressing perturbation dynamics have key importance in drug design.

A number of studies suggests that the representation of signaling pathways through Boolean Networks (BNs) may be a useful first step in this direction [19–23]. BNs became very popular for modeling signaling networks since the emergence of public databases (like KEGG [24]). This knowledge can be used to construct a BN of a biological process in which nodes are genes and edges represent interactions among them. A model can be obtained by associating a Boolean value to each node (e.g. 0 or 1 if the node is active or inactive) and a logic relation to each edge (i.e. AND, OR, NOT depending on the type of interaction between two nodes). Moreover, for simulation purposes BNs can be converted into ODEs with continuous time description, thus providing more understandable information about general pathway dynamics under different conditions. To support these studies, publicly available software tools such as BooleanNet [19], PATHOLOGIC-S [25] and Odefy [23] have been developed; they offer a scalable Boolean framework for modeling cellular signaling.

**Fig 1:**
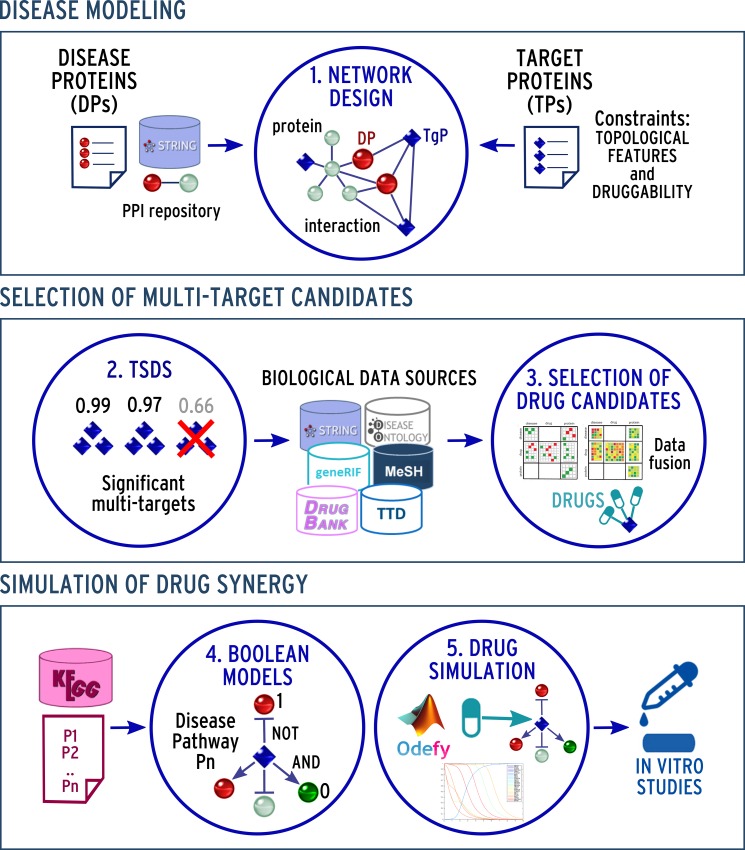
Overview of the proposed approach. (1) A PPI network is constructed starting from a list of disease proteins (DPs); then a list of target candidates (TPs) for drug synergy is obtained based on topological network properties; (2) A score function, called Topolgical Score of Drug Synergy (TSDS), assigns a score to each combination of TPs allowing the selection of significant multi-target combinations; (3) TP combinations are further augmented through the application of a data fusion approach. Here, the integration of several data sources [26] allows to obtain a list of known and predicted drug-target interactions; (4) The biological pathways related to disease progression are extracted; the pathways are represented with Boolean Networks (BNs); (5) BNs are simulated taking into account drug activities to understand biological pathways alterations through different pharmaceutical interventions. Finally, *in vitro* studies to validate the ability of the method to propose potential therapies can be carried on taking into account the results obtained from the previous phases.

Following these considerations, in this work we present a novel bioinformatics pipeline that combines network-based approaches and BN to integrate data and knowledge and to propose new therapeutic strategies or novel therapeutic uses of already approved drugs for a specific multi-factorial disease. An overview of the proposed approach is shown in Fig 1.

The first step of the method consists in building a PPI network by integrating high-throughput experiments and PPI data; then, by analyzing this network, a ranked list of target combinations is obtained through the application of a methodology that has been previously developed by our group [12]. This method has been extended to automatically select interesting drugs. It should be noted that the search space is not limited to known drug-target interactions, but also, through the application of a novel data-fusion method [26], to predicted ones. Finally, we build Boolean models of the pathways with the strongest association with diseases, in order to evaluate and predict which drug combinations achieve the greatest degree of perturbation of the phenotype. To assess our results, *in vitro* experimental data can be produced to validate the computed predictions.

In this work, we illustrate our approach and its potential validity by applying it to the case of Triple Negative Breast Cancer (TNBC). TNBC is a heterogeneous and aggressive subclass of breast cancer that affects 15% of all breast cancer patients. TNBC has the poorest prognosis of all subtypes with rapid progression leading to mortality in younger patients. So far, there is no targeted therapy for TNBC and the only approved treatment option is chemotherapy; for its poorly understood biology and for its complexity TNBC is a suitable candidate for network-based modeling and multicomponent therapeutics, as well as for drug repositioning. The developed approach extracted a number of drug candidates for repurposing, ranking their potential combinations, allowing to plan and execute *in vitro* experiments. In the results section, we will describe the findings we obtained, as well as the outcomes derived from *in vitro* testing the drugs. Results suggest that our method has the ability to select potential drug candidates and to provide potentially useful therapeutic strategies.

## Materials and Methods

The implementation of the developed approach (depicted in Fig 1) can be divided into five main steps: (1) PPI network design; (2) Multi-target ranking through TSDS score; (3) Identification of drug candidates; (4) Boolean modeling of disease pathways; (5) Simulation of drug actions and planning of in vitro studies. In this paper we show the application of the method through the TNBC case, although it can be readily applied to other diseases. The approach has been implemented in Python and Matlab; the code is available upon request from the authors.

### TNBC network design

The initial step of the proposed approach focuses on constructing a disease-specific PPI network and the selection of drug targets.

#### Selection of Disease Proteins

Since our aim is to build a PPI network tailored to TNBC, the proposed approach starts with the selection of a list of genes and proteins known to have a role in the disease. The list was generated through the analysis of a recent mutational study where different high-throughput experiments from 104 cases of primary TNBC were performed [27]. The analysis of these data allowed to extract a list of the most significantly mutated and differentially expressed genes in TNBC. The proteins that are codified by such genes have been called *Disease Proteins* (DPs) (depicted with red circles in Fig 1) and they were assumed to be the final destinations of the therapeutic effect of a potential therapy. The method has thus been developed by assuming that a successful multi-target treatment should have an effect on all DPs.

#### PPI network construction

The PPI network related to TNBC was derived by extracting the PPIs stored in the STRING public repository [28]. In our PPI network, two proteins (network nodes) are linked (network edge) if an interaction between them is found in STRING. The set of network nodes include the initial DP list and their STRING interactors, while the set of edges is restricted to the most reliable STRING associations, i.e.: (i) the associations need to be derived from experimental and database evidence; (ii) the STRING confidence score has to be higher than 0.7 (i.e. only edges with a score higher than the 70th percentile of the STRING weight distribution are taken into account). The cut-off has been chosen considering that STRING curators suggest 0.7 as a reference value for high-confidence association [28]. The resulting network is a weighted graph, where the edge weights are proportional to the confidence scores.

#### Target Selection

Once DPs were identified and the network built, the next step consisted in the network targeting. Instead of selecting all the network nodes as possible targets, the nodes space was restricted to most interesting nodes from a pharmacological point of view, as reported in [12].

In detail, potential *Target Proteins* (TPs) (depicted with blue diamonds in Fig 1) are identified following three constraints: (i) hub nodes are discarded as potential TPs; (ii) bridging nodes are elected as TPs; (iii) a TP has to be druggable.

The first constraint involves hubs, i.e. nodes having a number neighbors higher than average. Beside their topological and functional significance in networks, hubs have special biological properties due to their central role in modular organization of the protein interaction network. In fact, if hubs are selectively attached, the network information transfer rapidly deteriorates. Because of this property, they are usually considered attractive drug targets [7]. However, hubs often also correspond to essential proteins, thus, their attack may cause adverse effects or it may result in increased toxicity [7,12,29]. For these reasons, we decided to discard such nodes as potential TPs.

The selection of hubs in the network have been performed by considering the top 20% highest degree nodes, as suggested in [30].

An alternative strategy consists therefore in targeting a set of proteins that locally have less impact than hubs, but that may provide a synergistic effect on a broad portion of the disease network. To this end, the second constraint involves bridging centrality (BR), a centrality measure that can discriminate bridging nodes, i.e. the nodes that are crucial to dispatch information to the network topological structures: such nodes usually have fewer neighbors than hubs, and are typically located between highly connected regions (i.e. network modules). Bridge nodes are therefore regulated by the nodes of different modules and this may lead not only to a lower toxicity but also to a higher therapeutic effect, since the higher BR(i), the more information flows through node *i*. This makes bridging nodes good drug target candidates: therefore, bridging centrality can be used to locate the key target proteins TPs. Bridging nodes in the network were identified as the nodes whose values were in the highest quartile of the bridging centrality. This threshold was suggested by Hwang et al. and it has been also confirmed by empirical studies on several real world network systems [31]. BR of a node *i* was calculated as proposed by Hwang et al. [31] following the equation:

BR(i)=BC(i)∙B(i) (1)

where B is the betweenness centrality and BC is the bridging coefficient, which measure the global and local features of a node, respectively. It has to be noticed that instead of standard B, Random Walk Betweenness Centrality (RWBC) [12] is used in Eq 1 because it takes into account all possible paths between two nodes and not only the shortest paths between nodes (as B does) [32]. On the other hand, the bridging coefficient of a node *i* is computed as:

$$BC(i)=\frac{D(i)}{\sum_{v\epsilon N(i)}\frac{1}{D(v)}}$$ (2)

where *D*(*i*) is the degree of node *i*, and *N*(*i*) is the set of neighbors of node *i*.

Finally, in order to take into account the druggability of bridging proteins, we selected as TPs only those with interacting chemicals in the STITCH [33] protein-drug repositories. Also in this case, we selected only the associations with high confidence (STITCH confidence score > 0.9). In addition, the list of druggable network nodes has been extended by using DrugBank [34].

#### Multi-target ranking through TSDS score

The subsequent step (see Fig 1) of the procedure focused on ranking the target combinations through the application of the Topological Score of Drug Synergy (TSDS), a score function that we have previously developed [12] (for details, see S1 Appendix). For computational and therapeutic compliance reasons, the multi-target approach has been restricted to combinations of target triplets. In detail, each combination of three TPs is ranked according to its TSDS, a topological index based on the shortest paths connecting the TPs and the network DPs. The TSDS also takes into account the network edge weights (i.e. favoring shortest paths with higher edge weights). Next, significant target combinations are selected through the construction of a null distribution and by applying a p-value threshold (p-value < 0.01, for details, see S1 Appendix).

#### Selection of drug candidates through data fusion

The third step of the proposed approach aims selecting potential drugs candidates (see step 3 in Fig 1). First, DrugBank [34] and Comparative Toxicogenomics Database (CTD) [35] are used to extract a list of approved drugs known to interact with the TPs. Second, this list is further augmented through the application of a novel data fusion approach based on matrix tri-factorization [26,36] (for details, see S2 Appendix).

This approach, which extends a strategy used in recommender systems, represents data sources through association matrices, e.g. disease-gene, drug-target, protein-protein. Such matrices are then jointly factorized through a product of low-rank matrix factors (corresponding to ''latent'' dimensions, i.e. meta-genes, meta-proteins). The factors are found by means of a suitable optimization algorithm that allows to take into account the entire set of matrices all at once. When factors are multiplied, the original matrices are reconstructed with a good approximation while generating predictions on potential new interactions [26]. The application of this procedure to our problem enables to detect new interaction pairs between drugs and selected targets by combining various data sources with the aim to reposition drugs used for other disease to the TNBC case.

In detail, as we can see from Table 1, we took into account: (i) Diseases; (ii) Drugs; (iii) Proteins and their relationships (see Table 1). Each knowledge source is represented through a data matrix. A data matrix can link objects of the same type (e.g. protein-protein interactions) or objects of different types (e.g. drugs and their targets). Relationships between objects of the same type *i* are defined by the constraint matrix Θ_i_, while the links between the objects *i* and *j* are represented through the relation matrix R_i,j_ (for details, see S2 Appendix). Such matrices are simultaneously factorized by the algorithm to reveal hidden drug-target associations. The drugs predicted to interact with the significant targets are finally merged with the known drug list extracted from DrugBank and CTD. Thanks to this procedure a final list of candidates for selected targets is obtained.

**Table 1:** Collection of data sources used for matrix tri-factorization, their size and number of edges.

Matrices	Associations	# Nodes	# Interactions	Data Sources
Θ_1_	disease-disease	6337	35201	DiseaseOntology [37]
Θ_2_	drug-drug	1196	11921	DrugBank [34]
Θ_3_	protein-protein	14250	431600	STRING [28]
R_1,3_	disease-protein	1844/13250	96157	GeneRIF [38]
R_2,1_	drug-disease	766/134	799	TTD [39]
R_2,3_	drug-protein	1338/3585	15153	DrugBank [34]

### Boolean modeling of disease pathways

**Fig 2:**
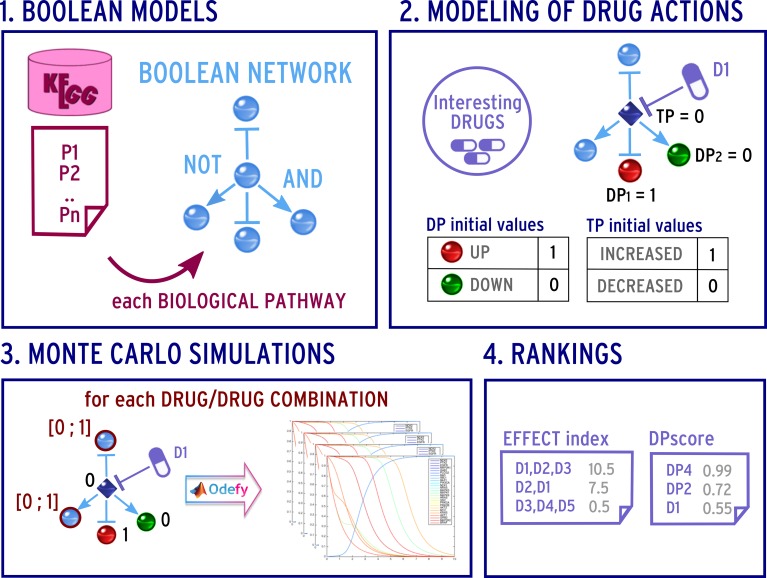
Predicting the drugs effect on biological pathways. (1) Boolean modeling of KEGG pathways; (2) Modeling the disease nodes and the pharmacological actions; (3) Monte Carlo simulations of the drug combination actions; (4) Ranking of the drug efficacy and the disease proteins.

After having selected the list of drugs, the proposed pipeline focuses on the construction of drug response prediction models, whose simulations should lead to the prediction of a single-compound drug efficacy as well as of a multi-compound drug efficacy. In our case study (as depicted in Fig 2), we concentrate our efforts on the signaling pathways related to the TNBC progression.

As a first stage (see step 1 in Fig 2), a list of biological pathways related to the TNBC progression is extracted from the KEGG pathways repository and each pathway is subsequently modeled as a Boolean Network (BN). To this end, we developed an automated procedure to process and convert KEGG signaling pathways into BNs. Each regulatory reaction in a pathway is translated into a logic formula by parsing the related KEGG KGML file related. In the KEGG database, interactions between molecules are mainly contained in signaling maps and they encode information such as “A activates B”. We translate every possible interaction into a Boolean function following the conversion rules shown in Table 2. For example, if in a pathway “A activates B” this relation has been automatically converted into “A AND B”. In this way, it is possible to assign a Boolean equation to every node belonging to a pathway. As a result, we obtain a BN for each TNBC-related pathway extracted from KEGG.

**Table 2:** Conversion table of KEGG associations into Boolean rules.

KEGG biological relationship	Symbol	Boolean rule
Activation	-->	AND
Inhibition	--|	NOT
Expression	-->	AND
Repression	--|	NOT
Indirect effect	..>	AND
State change	…	AND
Binding/association	--	AND
Dissociation	-+-	NOT
Phosphorylation	+p	AND
Dephosphorylation	-p	NOT
Glycosylation	+g	AND
Ubiquitination	+u	AND
Methylation	+m	AND

Next, further data were integrated into the BN models (see step 2 in Fig 2) in order to better specialize them to the TNBC case. In detail, we integrated gene expression microarray data about TNBC into the model: the TNBC database (TNBCDb) [40] was used to extract a list of differentially expressed genes (i.e. genes with fold-change (FC) values greater than 2 in TNBC tissue(s) versus different types of non-TNBC tissues). If a gene was differentially expressed in multiple experiments, it was considered up- or down- regulated by checking the FC values obtained in each experiment; i.e. its final differential expression direction is assigned based on the majority of times it was up- or down- regulated. If a gene was found both up- and down- regulated in the same number of experiments, it was discarded.

We then set the values of the BN nodes corresponding to these genes, i.e. Disease Nodes *DNs*, to 0 or 1 depending on their differential expression (i.e. down-regulation or up-regulation).

### Simulation of drug actions and planning of *in vitro* studies

In the final step of the proposed approach (see step 4 in Fig 1), the action of each interesting drug and drug combination was simulated in every selected pathway.

#### Initialization of target nodes

First, the administration of a drug or a drug combination was modeled by identifying the BN nodes corresponding to the related TP targets and by setting, as shown in Fig 2, their initial values to 0 or 1 based on the related drug action (i.e. 0 or 1 if the drug decreases or increases the expression or the activity of its targets, respectively). To this end, the Comparative Toxicogenomics Database [35] was used to exploit available knowledge on drug-gene interactions. In detail, we retrieved drug-gene interactions and their descriptions were analyzed in order to assign a final value (0 or 1) to the target genes of interesting drugs.

#### Monte Carlo Simulations of drug actions in pathways

As depicted in step 3 of Fig 2, since only the initial values of the DP and TP nodes were known, we performed *M* Monte Carlo simulations to randomly assign values to the other network nodes.

In detail, the simulations were performed using the MATLAB-toolbox *Odefy* [23]. Thanks to this procedure Boolean models are converted to continuous ODEs for a better visualization and interpretation of the node behavior (see plots in step 3 of Fig 2).

#### Drug combination ranking by EFFECT index

The simulation results were then analyzed to provide a ranking of drug therapies (see step 4 in Fig 2). To this end, we defined a global effect index, called the *EFFECT index*, which assigns a score to each selected drug and drug combination.

The *EFFECT index* can be computed in three main steps:

1. *PathEFF*_*MC*_ *index*. First, for each pathway we independently analyze the simulation results. Here, we assume that the therapeutic efficacy of a drug or a drug combination can be evaluated taking into account the BN nodes corresponding to Disease Nodes (*DNs*, see previous sections).
  A therapy is considered to be more effective than another one if it regularizes more *DNs*, while the other network nodes (i.e. not-disease nodes $\bar{DN}$) must not be perturbed by the drug action. Note that a node is considered a regularized node *RN* if its initial value changes after the simulation (i.e an up-regulated DP becomes 0 or a down-regulated DP becomes 1) otherwise it is called "not regularized" node ($\bar{RN}$). The identification of regularized nodes allowed us to assign a pathway-related score to every simulated treatment. This score, the *PathEFF* index, measures the potential effect of a treatment in a specific pathway. The *PathEFF* index is based on the construction of a confusion matrix, where:
  - True Positives (TrPs) correspond to the disease nodes *DN* correctly regularized by the drug administration.
  - False Positives (FPs) are network nodes not corresponding to *DNs*, whose values have been changed by the drug.
  - False Negatives (FNs) are *DN* nodes not regularized.
  - True Negative (TNs) are not-diseased nodes (i.e. $\bar{DN}$) not affected by the pharmacological action.
  The *PathEFF* of a drug or a drug combination *D* in a pathway *p* is then calculated as:
  $$PathEFF(D,p)=2∙\frac{precision∙recall}{precision+recall}$$ (3)
  where:
  $$precision=\frac{TrP}{TrP+FP}$$ (4)
  and
  $$recall=\frac{TrP}{TrP+FN}$$ (5)
  Precision and recall are calculated based on the confusion matrix of the considered pathway *p*. It is easy to see that the *PathEFF* index is the F-measure of the confusion matrix: the score is high if the proportion of disease genes that have been regularized (i.e. True Positive) in a pathway by a given drug administration is high.
  Following this procedure, the actions of each possible treatment were simulated in every selected pathway. Since we perform *M* Monte Carlo simulations (usually with M = 1000) for each drug *D* in each pathway *p*_*j*_, we average *PathEFF* over all the simulations:
  $${PathEFF}_{MC}\;(D,p_{j})=\frac{1}{M}\sum_{i=1}^{M}{PathEFF}_{i}$$ (6)
2. *DrugEFF index*. The individual *PathEFF*_*MC*_ indices are then averaged over all the pathways to obtain a global score of a treatment effect on the disease, i.e. the *DrugEFF index*. Formally, *DrugEFF* for a drug/drug combination *D* can be defined as:
  $$DrugEFF=\sum_{j=1}^{n}{PathEFF}_{MC}(D,p_{j})$$ (7)
  where *n* is the number of pathways in which at least one target of the drug *D* is included.
3. *EFFECT index*. Finally, in order to evaluate the effective potential activity of a treatment, the *DrugEFF* index can be compared to the *noDrugEFF* index, i.e. a measure of the pathway behaviors without treatment. This index is obtained by following the same procedure used to compute the *DrugEFF* index, except that the target TP values are not initialized.
  In this way, an *EFFECT index* of a drug or a drug combination *D* can be calculated as:
  $$EFFECT(D)=100∙\frac{DrugEFF(D)-noDrugEFF(D)}{noDrugEFF(D)}$$ (8)
  The *EFFECT(D)* can be used to rank therapies and to select the best candidates for *in vitro* studies.

#### Disease Protein ranking to support in vitro studies

Finally, a ranked list of DPs that should be monitored during *in vitro* experiments can be obtained by analyzing the results and taking into account the number of regularized DNs in a disease pathway.

The aim of this final procedure is to plan *in vitro* studies measuring the expression of a fixed panel of genes under different drug actions, in order to validate the ability of the method to retrieve potential therapies.

The ranked list can be obtained thanks to the definition of a score that evaluates, for each candidate combination (*drugComb*), the probability *P*_*k*_(*DP*) that a DP will be regularized in a pathway *k* in M Monte Carlo simulations. The probability *P*_*k*_(*DP*) can be obtained as:

Pk(DP)={Nr(DP)M0ifDPbelongstothepathwaykotherwise (9)

Where *Nr*(*DP*) is the number of times a DP is regularized in M simulations.

Next, the global score of a DP for a *drugComb* is computed as:

$$\begin{array}{c} {DP}_{score}=median[P_{1}\;(DP),{\ldots ,P}_{k}\;(DP),\ldots ,P_{N\;}\;(DP)]\\ if\;P_{k}(DP)\neq 0;\;N=number\;of\;pathways \end{array}$$ (10)

The median has been chosen in order to favor nodes that obtained high scores even if they do not belong to many pathways.

The final panel of DP is provided by computing a “consensus” ranking, taking into account the individual ranking preferences of the different drug combinations. To this aim, we used the Borda count, a form of preferential voting where rankings are converted into points, and the candidate that receives the most points is declared the winner [41].

## Results

**Fig 3:**
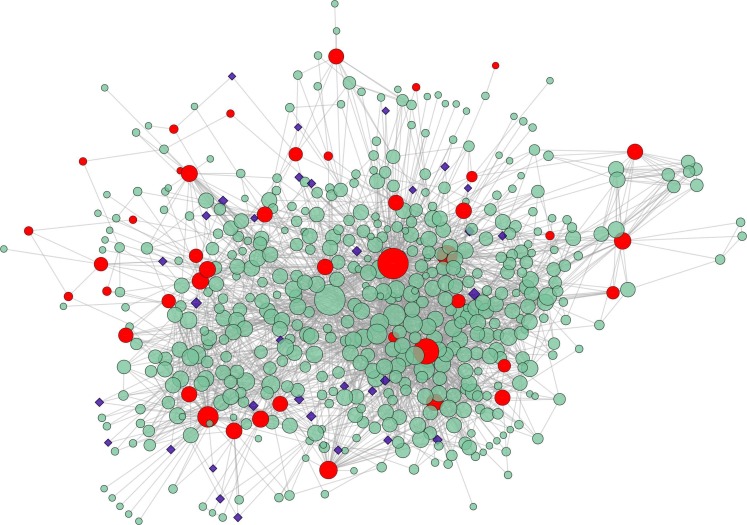
TNBC PPI Network. In the network the 43 DP seed nodes are highlighted in red while the 33 TP nodes are depicted by blue diamonds. The node size depends on the Bridging Centrality values as shown in the graph below the figure.

We started from the selection of a list of proteins known to be strongly related to the genetic variants caused by the disease. Analysis of the experimental data reported in [27] allowed to extract a list of 43 DPs codified by the most significantly mutated and differentially expressed genes in TNBC (see S1 Table). The list of DPs obtained with this procedure allowed building a PPI network by using the STRING database. The resulting TNBC network, shown in Fig 3, has 554 nodes (proteins) and 2602 edges (associations).

**Fig 4:**
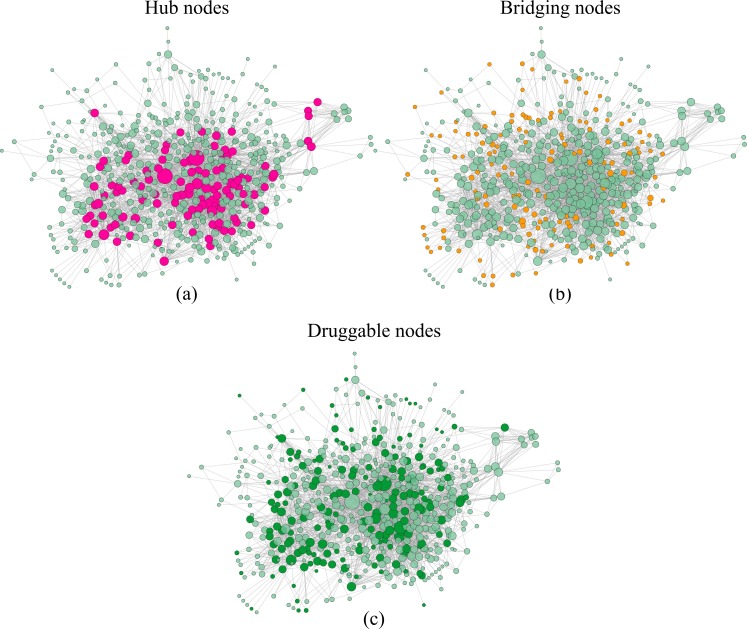
Network constraints to select TP nodes. In Fig 4(a) hubs are highlighted in pink. Note that these nodes are discarded as potential TPs. In Fig 4(b) orange nodes correspond to the bridging nodes, while in Fig 4(c) druggable nodes are depicted in dark green. The node size is proportional to its degree (i.e. number of neighbors).

We then selected target protein (TP) candidates according to the approach described above and in [12]. We identified 110 hub nodes by selecting the top 20% of nodes ordered by their degree values (depicted in pink in Fig 4(A)) and 139 bridging nodes by considering only the proteins in the highest quartile (0.003087) of bridging centrality values (reported as orange nodes in Fig 4(B)). Bridging nodes were selected as TPs. Finally, in order to evaluate druggability, the use of DrugBank and CTD allowed the extraction of 6074 drugs or compounds associated to 180 network nodes. Only such nodes, depicted in dark green in Fig 4 (C), were considered as druggable and thus as potential targets.

The final list contains 33 TP nodes, selected out of 110 hub proteins (discarded), 139 bridging and 180 druggable nodes. They are listed in Table 3 and depicted in blue diamonds in Fig 3.

**Table 3:** List of network Target Proteins TP. The column Freq. reports the protein frequency in the significant triplets.

Ensembl Prot ID	Gene Name	Description	Frequency
ENSP00000400175*	RHOA	ras homolog family member A	68
ENSP00000344220*	PDPK1	3-phosphoinositide dependent protein kinase 1	57
ENSP00000261584*	PALB2	partner and localizer of BRCA2	38
ENSP00000380024*	ING4	inhibitor of growth family, member 4	38
ENSP00000302564*	BCL2L1	BCL2-like 1	32
ENSP00000324173*	HSPA5	heat shock 70kDa protein 5	29
ENSP00000295400*	TGFA	transforming growth factor, alpha	25
ENSP00000364929*	ING1	inhibitor of growth family, member 1	21
ENSP00000265171*	EGF	epidermal growth factor	18
ENSP00000262033*	PTGES3	prostaglandin E synthase 3 (cytosolic)	14
ENSP00000262948*	MAP2K2	mitogen-activated protein kinase kinase 2	14
ENSP00000302886*	PA2G4	proliferation-associated 2G4, 38kDa	11
ENSP00000276603*	TERF1	telomeric repeat binding factor (NIMA-interacting) 1	10
ENSP00000291700*	S100B	S100 calcium binding protein B	8
ENSP00000361275*	PLK3	polo-like kinase 3	6
ENSP00000381098*	GRIP1	glutamate receptor interacting protein 1	1
ENSP00000005257	RALA	v-ral simian leukemia viral oncogene homolog A (ras related)	0
ENSP00000233057	EIF2AK2	eukaryotic translation initiation factor 2-alpha kinase 2	0
ENSP00000238721	TP53I3	tumor protein p53 inducible protein 3	0
ENSP00000264818	TYK2	tyrosine kinase 2	0
ENSP00000270279	CBLC	Cbl proto-oncogene C, E3 ubiquitin protein ligase	0
ENSP00000278385	CD44	CD44 molecule (Indian blood group)	0
ENSP00000316032	NUP98	nucleoporin 98kDa	0
ENSP00000321410	MAPK9	mitogen-activated protein kinase 9	0
ENSP00000326031	PPP1CA	protein phosphatase 1, catalytic subunit, alpha isozyme	0
ENSP00000338799	IL6ST	interleukin 6 signal transducer	0
ENSP00000342924	MCPH1	microcephalin 1	0
ENSP00000347046	PDE5A	phosphodiesterase 5A, cGMP-specific	0
ENSP00000356529	RGS16	regulator of G-protein signaling 16	0
ENSP00000357283	LMNA	lamin A/C	0
ENSP00000369981	SH3GL2	SH3-domain GRB2-like 2	0
ENSP00000370330	ERBB2IP	erbb2 interacting protein	0
ENSP00000379330	NFATC2	nuclear factor of activated Tcells, cytoplasmic, calcineurin-dependent 2	0

*Proteins resulted in significant combinations are marked with an asterisk.

The TSDS for each possible combination of 3 targets was then calculated and a null distribution was computed to find the significant TSDS proteins. In detail, the most significant triplets with p-values<0.01 resulted in 134 combinations of 16 different involved TPs. The significant target nodes are marked with an asterisk in Table 3; the table also reports their frequency in the triplets.

The third step involves the selection of drug candidates to be repurposed to the TNBC.

We first retrieved 7 approved drugs with known significant drug-target interactions from DrugBank and CTD repositories. Second, by applying the tri-factorization algorithm, we identified 8 predicted drugs associated to network nodes out of 816 predicted drug-target associations. The known and predicted drugs and the related significant TP nodes are listed in Table 4.

**Table 4:** Known and predicted drugs associated with significant TP nodes.

Significant TP	GeneName	Known Drug	Predicted Drug
ENSP00000344220	PDPK1	Celecoxib	No drugs
ENSP00000302564	BCL2L1	No drugs	Imatinib*, Hydroxyurea*, Azacitidine*,L-Aspartic Acid*, Flucytosine*
ENSP00000324173	HSPA5	Antihemophilic Factor	No drugs
ENSP00000265171	EGF	Sucralfate	No drugs
ENSP00000262948	MAP2K2	Bosutinib, Trametinib*, Vemurafenib*	Mercaptopurine, Dimethyl fumarate, Carbidopa
ENSP00000291700	S100B	Olopatadine	No drugs

*Drug selected as promising candidates are marked with an asterisk.

Within the drug lists, 7 promising drugs, i.e. Imatinib, L-Aspartic Acid, Vemurafenib, Hydroxyurea, Azacitidine, Flucytosine and Trametinib, have been selected for further investigations. In fact, they seem to be suitable repositioned in TNBC, since previous studies indicate their effective use in malignancies other than breast cancer [42–48]. A particularly interesting candidate that emerged using our method was Imatinib. Imatinib is a well-described protein tyrosine kinase inhibitor that has potent activity against the oncogene fusion protein, BCR-ABL, the platelet-derived growth factor receptor (PDGFR), and the growth factor receptor of the tyrosine kinase subclass III family, C-Kit (or CD117). The clinical activity of Imatinib was first established in the treatment of chronic myelogenous leukemia (CML), a disease defined by the overexpression of BCR-ABL. When administered to patients with CML, the response rate (RR) to Imatinib was over 90%, with most patients experiencing long-term disease control [42]. Imatinib is also indicated for the treatment of gastrointestinal stromal tumor (GIST) in which C-Kit is typically overexpressed, resulting in a disease control rate of over 80% [49]. Moreover, interesting data have suggested that imatinib can induce a positive response in at least 90% of patients with dermatofibrosarcoma protuberans (DFSP) [50]. Imatinib has limited efficacy as a single agent in conditions where overexpression of its tyrosine kinase target has not been well defined. However, some preliminary experiences of Imatinib in combination with other cytotoxic agents, reported a sort of anti-tumor activity, even in the absence of specific molecular targets, in different types of solid malignancies. In metastatic breast cancer the use of combination of chemotherapy (i.e. Vinorelbin, Taxanes or Capecitabine) with Imatinib derived a limited but measurable clinical benefit, in heavily pre-traeted mBC patients [51].

From above and with the intent to deeply investigate the off-targets effect of Imatinib, based on the suggestion of our model, we decided to focus our further analysis on the combinations involving this drug.

Following the pipeline, we extracted from the KEGG repository a list of 18 pathways (detailed in Table 5) mostly involved in the TNBC progression and related to the targets of the selected drugs. Each pathway was then modeled as a Boolean Network (BN). The numbers of nodes, edges and disease proteins of the pathways are provided in Table 5.

**Table 5:** Pathways selected from KEGG. For each pathway, the number of nodes and edges of the related BN as well as the number of DPs and drug targets (for each of the drugs considered) present in the pathway networks are listed. In the table *No TPs* means that no drug targets were found in the pathway.

KeggID	Pathway Name	#Nodes	#Edges	#DPs	Imatinib	Vemurafenib	Flucytosine
hsa04062	Chemokine	56	85	21	9	3	*No TPs*
hsa04060	Cytokine-cytokine	236	217	50	15	*No TPs*	*No TPs*
hsa04012	ErbB	57	113	21	12	3	*No TPs*
hsa04068	FoxO	76	115	26	8	3	*No TPs*
hsa04066	HIF-1	73	106	21	15	1	1
hsa04910	Insulin	69	104	18	11	3	*No TPs*
hsa04630	Jak-STAT	30	47	9	7	*No TPs*	*No TPs*
hsa04010	MAPK	128	226	40	15	4	1
hsa04150	mTOR	43	63	12	12	1	No TPs
hsa04115	p53	57	98	16	7	*No TPs*	2
hsa05200	Pathways in cancer	146	274	56	33	3	3
hsa04151	PI3K-Akt	83	140	28	18	3	3
hsa04015	Rap1	78	109	23	5	4	*No TPs*
hsa04014	Ras	80	135	25	7	4	*No TPs*
hsa04350	TGF-beta	47	62	13	5	*No TPs*	*No TPs*
hsa04668	TNF	50	90	16	15	1	*No TPs*
hsa04620	Toll-like	72	124	23	15	1	*No TPs*
hsa04370	VEGF	33	51	14	7	3	*No TPs*

**Fig 5:**
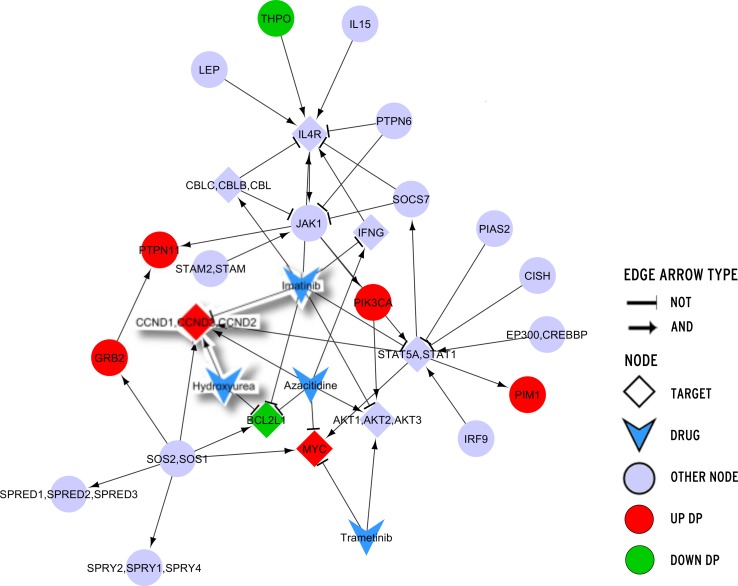
Boolean Network of the Jak-STAT signalling pathway.

We then extracted from the TNBC database (TNBCDb) [40] a list of differentially expressed genes. The list included 2052 up-regulated and 1239 down-regulated genes. For each BN related to a KEGG pathway, the initial values of the DPs were automatically assigned according to their expression values (i.e. 0 for down-regulated genes and 1 for up-regulated genes; see green and red nodes in Fig 5). S2 Table reports the 111 up-expressed and the 62 down- expressed genes belonging to at least one of the selected pathways.

Before running the BN simulation *Odefy*, the possible drug combinations were restricted to the ones whose actions are not opposite on their shared targets (e.g. a drug pair is discarded if one drug activates a target that is inhibited by the other drug). For example, looking at the network shown in Fig 5, a combination of Hydroxyurea and Imatinib was discarded because of their opposite interaction with CCND1, CCND2, CCND3 genes.

Under this assumption, we considered the following combinations: Imatinib, Imatinib-Vemurafenib, Imatinib-Flucytosine and Imatinib-Vemurafenib-Flucytosine.

For each selected drug, we extracted the related TP targets by using CTD: the numbers of targets participating in each KEGG pathway for the selected drugs are shown in Table 5. As evident in the table, Imatinib targets are involved in all the disease pathways, unlike the other drug’s targets. Afterwards, we simulated the action of every combination in each pathway by fixing the initial values of the TP involved in the pathway and associated with the drugs in the combination. Initial values were set to 0 or 1 based on CTD interactions between the TPs and the drug candidates.

**Fig 6:**
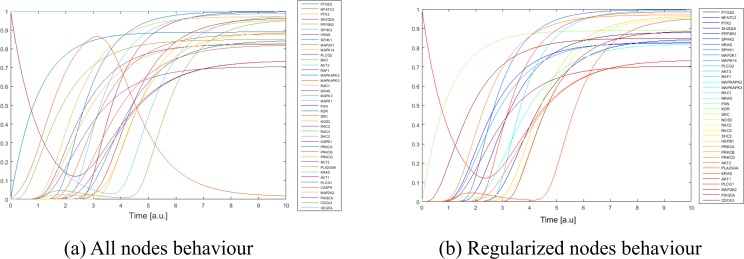
An example of Odefy outputs obtained by simulating Imatinib administration in Jak-STAT signaling pathway.

By applying the Odefy toolbox with 1000 Monte Carlo simulations, we simulated the action of Imatinib and drug combinations involving Imatinib in order to estimate the behaviors of the selected 18 biological pathways. An example of the output is provided in Fig 6: Fig 6(A) shows all gene nodes behaviors after the Imatinib action simulation, while Fig 6(B) reports only the ones related to the regularized nodes.

**Fig 7:**
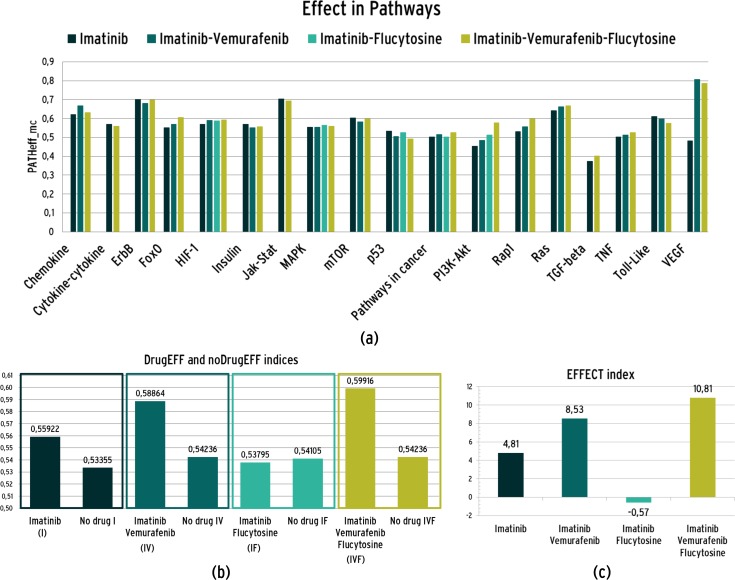
(a) PathEFF_MC_ index for each simulated treatment in every pathway; (b) DugEFF(D) and the related noDrugEFF(D) for each drug combination; (c) *EFFECT* index for each simulated drug administration

The effects of drug treatments were then evaluated for all possible drug combinations in each pathway by computing the *EFFECT* index. First, the *PathEFF*_*MC*_ index was calculated as in Eq (6) to obtain a score of the effect of each simulated therapy in all disease pathways. The results are shown in Fig 7(A) and details on the data are provided in S3 Table.

The *DrugEFF* index was then calculated for all the 4 treatments following Eq (7). Here, the *DrugEFF* index for the combinations was computed by averaging only the *PathEFF*_*MC*_ of the pathways involving at least one TP of Vemurafenib or Flucytosine. With the same procedure, for each treatment, the related *noDrugEFF* indices were calculated to evaluate the behaviors of untreated pathways. The resulting *DrugEFF* and *noDrugEFF* are shown in Fig 7(C). Lastly, the final therapy *EFFECT* index, computed as in (8), allowed capturing the differential effect on treated and untreated pathways (see Fig 7(C)). In detail, Imatinib had a positive effect on the treatment of the disease (EFFECT = 4.81), as well as a combination of Imatinib and Vemurafenib (EFFECT = 8.53) and a combination of Imatinib and Vemurafenib and Flucytosine (EFFECT = 10.47). On the other hand, Imatinib combined with Flucytosine had a small negative effect (EFFECT = -0.57).

Finally, we calculated the *DP*_*score*_ (according to Eq (10)). This procedure provided a ranked list (see S4 Table) of DPs that were suggested to be monitored during *in vitro* experiments.

### *In vitro* results

According to the previous findings, *in vitro* experiments were carried out to validate the proposed approach and to verify the potential effectiveness of the selected combinations.

**Fig 8:**
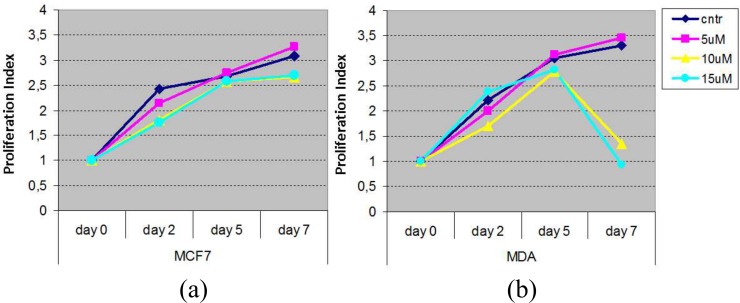
Evaluation of cell viability performed by treating MCF7 and MDA-MB-231 cell lines with different doses of Imatinib. MCF7 cell line is taken as control, while MDA-MB-231 is used as representative of TNBC.

As a first stage, an MTT assay was performed to assess how different cell lines react under different concentrations of Imatinib. In detail, the MCF7 cell line of luminal breast cancer subtypes was used as a control line, while the MDA-MB-231 cell line was taken as representative of TNBC. Cell viability was evaluated in MCF7 and MDA MB 231 cells after 2-5-7 day of treatment with different concentration of Imatinib (5, 10, 15 μM; Sigma). The number of viable cells was detected using CellTiter Aqueous Assay kit (Promega Corporation) accordingly to manufacturer's instructions. Untreated MCF7 or MDA-MB-231 cells were used as a control for cell death measurement. The effects of Imatinib administration at different doses and times are shown in Fig 8 (for details, see S5 Table).

**Fig 9:**
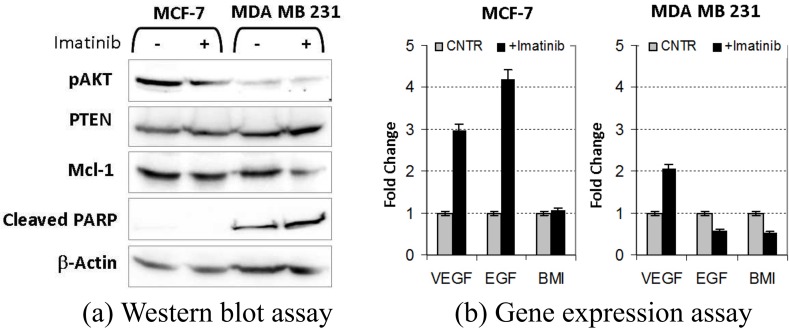
Disease genes evaluations by treating disease and control cell lines with Imatinib.

An evaluation of the behavior of interesting DPs was performed after a one-week treatment with 10 μM Imatinib on both cell lines (see Fig 9 (A)) with Western blotting experiment. Here, SDS PAGE was performed as previously described in [52]. Primary antibodies were: mouse monoclonal cleaved-PARP (1:100, Santa Cruz), Mcl1 (1:100, Santa Cruz), beta-actin (1:200, Santa Cruz), or rabbit polyclonal pAKT (1:400, Cell Signaling technology).

Finally, a gene expression assay of the relevant DPs was performed by using a quantitative real-time PCR (qPCR). In these experiments, total RNA of each sample was isolated and cDNA achieved using High Capacity cDNA Archive kit as recommended by manufacturer (LifeTech). For amplification 50ng of cDNA/sample was combined with specific primers (TaqMan Assay) as recommended by manufacturer (LifeTech). Amplification was performed by Vii7 Real-Time PCR systems (LifeTech). All amplification reactions were performed in triplicate, and the relative quantitation of genes expression was calculated using the comparative Ct method (DeltaDeltaCt). Glyceraldehyde-3-phosphate dehydrogenase (GAPDH) was used as endogenous control. Data processing and statistical analysis were performed using Vii7 software. The results are shown in in Fig 9(B).

As predicted by our approach, TNBC cell lines show increased sensitivity to Imatinib in comparison with luminal-like breast cancer cells.

**Fig 10:**
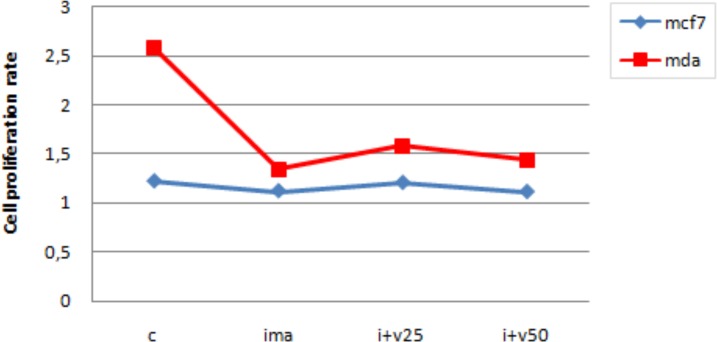
Evaluation of proliferation rate of TNBC cells (MDA-MB-231) and luminal-like breast cancer cells (MCF7).

In addition, the method predicts a further synergic cytotoxic effect when TNBC cell lines are treated with Imatinib and Vemurafenib. To establish this synergy, we evaluated the proliferation rate of TNBC cells and luminal-like breast cancer cells treated with Imatinib 10 μM and Vemurafenib 25 μM for three days. The results are shown in Fig 10. Fig 10 also shows the proliferation rate obtained by treating the cell lines only with Imatinib (see ima on the x axis).

## Discussion

Cancer clinical trials have been the key for the major advances in modern oncology. However, in the last years a clear recognition about the obstacles of such a model of clinical research emerged within the academic, and pharmaceutical/biotech communities, with the evidence that our current conception of new drug discovery and development is no longer fit for purpose. Minimal success rates of drug approvals, poor safety profiles, and long development processes are some of many hurdles encountered in the drug discovery. In this scenario, drug repurposing can provide an alternative approach, to meet the demands of the new, potent and safe anticancer agents in terms of both economic cost and time efficiency. The common molecular pathways of different diseases and secondary indications of most of the approved drugs, together with advances in genomics, informatics and biology, and finally with the availability of approved or safe drug libraries can provide an efficient way of screening safer drugs for new indications. Network based bioinformatics approaches, able to integrate the best knowledge on a specific disease and relevant drugs information, open new avenues for an effective pre-analytical screening that could be incorporated in the challenging process of anti-cancer drug discovery.

In our work, we have investigated the application of our proposed framework to the case of TNBC, a subclass of breast cancer that still doesn't have clearly identified molecular targets.

For TNBC, the PPI network was constructed starting from a list of proteins derived from the analysis of a recent mutational study on TNBC [27].

The targets selected as candidates by applying the TSDS function on TNBC network are shown in Table 3 and, interestingly, none of them is currently used in robust ongoing clinical trials, thus opening the study to new prospects for TNBC treatments. Moreover, the drugs we identified as potential candidates (e.g. Imatinib, L-Aspartic Acid, Vemurafenib, Hydroxyurea, Azacitidine, Flucytosine and Trametinib) seem to be suitable repositioned in TNBC since their effect in other malignancies have been already assessed [42–48].

As anticipated, a particularly interesting candidate that has been predicted using our method is Imatinib, the elective drug for chronic myeloid leukemia and GISTs, and whose off-targets activity herein identified, suggest encouraging antitumor opportunities in TNBC.

The use of BNs allows for a better interpretation of molecular patterns of tumors under different drug administrations. Currently there are many approaches to model and simulate biological processes, such as signalling pathways (e.g. Petri networks, Bayesian networks, differentially equations ODEs [53]) that can be considered as valid alternatives. We decided to choose BNs since they provide a simple and intuitive representation of the biological interactions occurring between genes and they are amenable to easily represent KEGG pathways. Moreover, thanks to the Matlab tool *Odefy* it has been possible to convert them into continuous models and obtain a visual representation of the node response to external stimuli (i.e. drug action). BNs are clearly a strong simplification of the real biological pathways behaviors, but they are considered good mathematical models able to predict ordered sequences of activation/inhibition patterns, albeit without predicting the exact dynamics of a biomolecular network. In our case, their use is instrumental to highlight the pathways that can be investigated first to verify the efficacy of the proposed drug candidates.

The construction and simulation of BNs for the pathways mostly related to TNBC progression under drug activities confirmed that Imatinib is a suitable candidate for TNBC treatment. Many of its targets are involved in all the pathways related to TNBC (see Table 5). On the other hand, the other drugs selected to be combined with Imatinib have fewer targets participating in such pathways.

Moreover, analysis of the simulation results performed through the definition of *EFFECT* index allowed to clarify whether a stronger effect can be achieved by combining Imatinib with other drugs, such as Vemurafenib and Flucytosine. The results showed that the predicted stronger effects on the disease are obtained by combining Imatinib with Vemurafenib or both with Vemurafenib and Flucytosine (see Fig 7). On the contrary, the combination of Imatinib and Flucytosine resulted in a negative EFFECT index and, therefore, such multi-drug treatment has been discarded for future experiments.

Based on the results we obtained, different *in vitro* studies have been performed to validate the ability of our approach to retrieve potential therapeutics. First, we performed an MTT assay to assess how TNBC cell lines reacted under different concentrations of Imatinib. Remarkably, the curve decreased (see Fig 8) only for the TNBC cell line but not for the controls, demonstrating the drug selectivity for the tumor subtype.

The results of the evaluation of interesting DP signaling (shown in Fig 9 (A)) confirmed that Imatinib can be considered as a potential drug candidate, since proteins related to a decrease in survival such as PTEN and Mcl-1 showed decreased activity in the treated TNBC line, while proteins such as Cleaved PARP, related to DNA damage, showed an increased signal (see Fig 9 (A)).

Finally, the results of a gene expression assay of the relevant DPs demonstrated a decreased expression of EGFR and BMI only in the TNBC line (see Fig 9 (B)). These two genes are involved in TNBC progression, again supporting the potential activity of Imatinib.

Preliminary *in vitro* experiments were also performed on TNBC and control cell lines using a combination of Imatinib and Vemurafenib. As shown in Fig 10, we found a significant delay in the proliferation of TNBC cells in comparison with luminal-like breast cancer cells, due to a synergic cytotoxic effect of the two drugs. Thus, a lower proliferation rate in the TNBC cell lines was found when using a multi-drug approach instead of the single drug treatment. This finding confirms that synergistic treatments may have a higher impact on complex diseases such as TNBC.

This work has shown the applicability of a network-based approach to identify potential drug targets by integrating specific molecular profiles of a disease subclass with data and knowledge extracted from a variety of information sources.

The developed methodology can easily be applied to a large variety of multifactorial diseases thus enabling the selection of tailored treatments, one of the crucial components of precision medicine.

For instance, if we are interested in investigating therapies for a specific patient, the PPI network can be constructed on the basis of the list of mutated proteins in the patient's specific proteomic or genomic background.

The results obtained by applying the proposed approach can also be translated into practical recommendations for planning *in vitro* experiments aimed at validating the identified drug candidate combinations.

Future directions of this work will be devoted to address its current limitations, both from the experimental and the methodological perspectives.

As concerns the experimental aspects, additional validation and testing of some of the presented procedures will be included. Other in vitro studies will be performed to further investigate the Imatinib-Vemuafenib combination. Moreover, the triplet of Imatinib, Vemurafenib and Flycitosine will be tested, too, in order to validate the findings obtained about its potential effect on TNBC.

As relates to the methodological aspects, more detailed models of disease pathways will be studied, in particular Bayesian networks and Petri networks, in order to get a more detailed description of the biological processes involved in the disease and in drug activities and of their synchronization. In particular, an actual restriction introduced by using Boolean networks is that such models allow the representation of the problem with binary variable and logic relations. In contrast, Bayesian networks may allow representing the problem with multiple states. Therefore, in the network modeling, it will be possible to include information about the differential expression of specific genes (i.e. how much a gene is over- or under- expressed), the normal states and the activity of drug combinations that have opposite effects on certain targets. Moreover, Petri Networks may allow better representing control/synchronization mechanisms and to manage inconsistent and incomplete data [54].

A future extension of the proposed approach can be aimed at the integration of available knowledge regarding side-effects. A possible way to select further interesting candidates is to investigate drugs having side effects similar to the ones identified by the proposed method [55–57]. To this aim, repositories such as SIDER can be used to retrieve drug side-effects [58]. Another option could be to include the side-effect information in the trifactorization algorithm thus highlighting potential novel drug–target interactions by taking into account such knowledge. This would be possible by adding a constraint drug-drug matrix representing drug similarities based on the number of shared side effects.

Finally, other drug-protein repositories can be exploited, such as STITCH, thus allowing considering also drugs derived from experiments, databases and the literature [33], together with approved ones.

## Conclusion

The development of an efficient bioinformatics framework is definitely needed to support drug design in order to automatically provide or repurpose tailored therapies for a specific disease or for a group of patients. However, it is difficult to systematically investigate the molecular mechanisms underlying a complex disease, such as cancer, due to its multifactorial nature.

Recently, network-based approaches have been largely used to integrate, analyze and visualize the available knowledge on a disease. Moreover, combining different types of information (e.g. drug interactions, biological signaling pathways) into network models may help to better understand the molecular mechanism of drug actions and to investigate potential drug therapies.

In this work, we presented a network-based framework for a feasible and efficient identification of personalized drug treatments that may be used to deal with complex diseases, such as cancer. Such framework is specifically designed to combine drug repurposing and multi-target therapy.

We propose a generic pipeline to rank multi-target proteins through the definition of the TSDS score based on network analysis. The fusion of different data and knowledge sources by applying the tri-factorization algorithm allows exploring the possibility of drug repurposing for the disease under study. Simulations of drug administration by modeling biological pathways into Boolean networks helped to provide suggestions for testing *in vitro* the most promising candidates. The experimental validation demonstrated the ability of the proposed approach to reveal potential thera*pies.

## Supporting Information

S1 Table List of Disease Proteins Dps. (DOCX)

S2 Table Differentially expressed genes in TNBC. This information was used to assign the BN initial values to DP nodes according to their differential expression.
(DOCX)

S3 Table Indices of efficacy of each drug combination. I: Imatinib, I+V: Imatinib combined with Vemurafenib, I+V: Imatinib combined with Flucytosine and I+V+F: Imatinib, Vemurafenib and Flucytosine combined all together. Note that no TP can occur when the drug used in combination with Imatinib does not have any target in the pathway. In the last rows the DrugEFF and the EFFECT indices are provided.
(DOCX)

S4 Table Borda score to rank DP genes that can be elected to be monitored in in vitro experiments. (DOCX)

S5 Table Results of cell viability performed by treating MCF7 and MDA-MB-231 cell lines with different doses of Imatinib. (DOCX)

S1 Appendix TSDS score. (DOCX)

S2 Appendix Data fusion approach. (DOCX)
