# Correction: A Network-Based Data Integration Approach to Support Drug Repurposing and Multi-Target Therapies in Triple Negative Breast Cancer

**DOI:** 10.1371/journal.pone.0170363

**Published:** 2017-01-11

**Authors:** Francesca Vitali, Laurie D. Cohen, Andrea Demartini, Angela Amato, Vincenzo Eterno, Alberto Zambelli, Riccardo Bellazzi

In the methods section, within the sub section titled Target Selection, there is an error in Formula (2). Please see the correct Formula (2) here.

$$BC(i)=\frac{1/D(i)}{\sum_{v\in N(i)}\frac{1}{D(V)}}$$ (2)

## References

[1] Vitali F, Cohen LD, Demartini A, Amato A, Eterno V, Zambelli A, et al (2016) A Network-Based Data Integration Approach to Support Drug Repurposing and Multi-Target Therapies in Triple Negative Breast Cancer. PLoS ONE 11(9): e0162407 doi:10.1371/journal.pone.0162407 27632168 10.1371/journal.pone.0162407 PMC5025072

