# Supplementary material for: A Network-Based Data Integration Approach to Support Drug Repurposing and Multi-Target Therapies in Triple Negative Breast Cancer
Source: PLoS One. 2016 Sep 15;11(9):e0162407. doi: 10.1371/journal.pone.0162407 (PMC5025072; doi:10.1371/journal.pone.0162407)
Supplement: S1 Table — (DOCX) [file pone.0162407.s001.docx]

S1 Table. List of Disease Proteins DPs.

| **Ensembl Protein ID** | **Gene Names** | **Description** |
| --- | --- | --- |
| ENSP00000391774 | CPSF6 CFIM68 | Cleavage and polyadenylation specificity factor subunit 6 |
| ENSP00000267101 | ERBB3 HER3 | Receptor tyrosine-protein kinase erbB-3 |
| ENSP00000263967 | PIK3CA | Phosphatidylinositol 4,5-bisphosphate 3-kinase catalytic subunit a |
| ENSP00000220959 | UBR5 EDD EDD1 HYD KIAA0896 | E3 ubiquitin-protein ligase UBR5 |
| ENSP00000288602 | BRAF BRAF1 RAFB1 | Serine/threonine-protein kinase B-raf |
| ENSP00000302530 | BUB1 BUB1L | Mitotic checkpoint serine/threonine-protein kinase BUB1 |
| ENSP00000231509 | NR3C1 GRL | Glucocorticoid receptor |
| ENSP00000336868 | CENPA | Histone H3-like centromeric protein A |
| ENSP00000353847 | WWTR1 TAZ | WW domain-containing transcription regulator protein 1 |
| ENSP00000269305 | TP53 P53 | Cellular tumor antigen p53 |
| ENSP00000367830 | PRKCZ PKC2 | Protein kinase C zeta type |
| ENSP00000290130 | MIS18A C21orf45 C21orf46 FASP1 | Protein Mis18-alpha |
| ENSP00000343204 | JAK1 JAK1A JAK1B | Tyrosine-protein kinase JAK1 |
| ENSP00000349156 | SIAH1 HUMSIAH | E3 ubiquitin-protein ligase SIAH1 |
| ENSP00000278616 | ATM | Serine-protein kinase ATM |
| ENSP00000342793 | PLD1 | Phospholipase D1 |
| ENSP00000288490 | DGKI | Diacylglycerol kinase iota |
| ENSP00000306124 | PRKCE PKCE | Protein kinase C epsilon type |
| ENSP00000247970 | PIN1 | Peptidyl-prolyl cis-trans isomerase NIMA-interacting 1 |
| ENSP00000358548 | NRAS HRAS1 | GTPase NRas |
| ENSP00000275493 | EGFR ERBB ERBB1 HER1 | Epidermal growth factor receptor |
| ENSP00000363092 | PRKG1 PRKG1B PRKGR1A PRKGR1B | cGMP-dependent protein kinase 1 |
| ENSP00000310551 | LCLAT1 AGPAT8 ALCAT1 LYCAT | Lysocardiolipin acyltran |
| ENSP00000267163 | RB1 | Retinoblastoma-associated protein |
| ENSP00000342056 | CS | Citrate synthase, mitochondrial |
| ENSP00000265944 | MYO3A | Myosin-IIIa |
| ENSP00000263857 | POLR1A | DNA-directed RNA polymerase I subunit RPA1 |
| ENSP00000369497 | BRCA2 FACD FANCD1 | Breast cancer type 2 susceptibility protein |
| ENSP00000263125 | PRKCQ PRKCT | Protein kinase C theta type |
| ENSP00000269571 | ERBB2 HER2 MLN19 NEU NGL | Receptor tyrosine-protein kinase erbB-2 |
| ENSP00000358414 | HMGCS2 | Hydroxymethylglutaryl-CoA synthase, mitochondrial |
| ENSP00000262848 | PRKX PKX1 | cAMP-dependent protein kinase catalytic subunit PRKX |
| ENSP00000343741 | ATR FRP1 | Serine/threonine-protein kinase ATR |
| ENSP00000309181 | CCNE2 | G1/S-specific cyclin-E2 |
| ENSP00000355865 | PARK2 PRKN | E3 ubiquitin-protein ligase parkin |
| ENSP00000361021 | PTEN MMAC1 TEP1 | Phosphatidylinositol 3,4,5-trisphosphate 3-phosphatase and dual-specificity protein phosphatase PTE |
| ENSP00000350719 | SYNE2 KIAA1011 NUA | Nesprin-2 |
| ENSP00000294016 | ADCY9 KIAA0520 | Adenylate cyclase type 9 |
| ENSP00000302955 | RRM2 RR2 | Ribonucleoside-diphosphate reductase subunit M2 |
| ENSP00000262367 | CREBBP CBP | CREB-binding protein |
| ENSP00000412292 | DEPDC1 DEPDC1A | DEP domain-containing protein 1° |
| ENSP00000299328 | TAZ | Tafazzin |
| ENSP00000265368 | SYNE1 | Nesprin-1 |
