# Supplementary material for: A Network-Based Data Integration Approach to Support Drug Repurposing and Multi-Target Therapies in Triple Negative Breast Cancer
Source: PLoS One. 2016 Sep 15;11(9):e0162407. doi: 10.1371/journal.pone.0162407 (PMC5025072; doi:10.1371/journal.pone.0162407)
Supplement: S2 Table — This information was used to assign the BN initial values to DP nodes according to their differential expression. (DOCX) [file pone.0162407.s002.docx]

S2 Table. Differentially expressed genes in TNBC. This information was used to assign the BN initial values to DP nodes according to their differential expression.

| **Expression** | **Genes** |
| --- | --- |
| Decreased  (initial_value = 0) | ADCY9, AR, AREG, ATM, ATR, BAD, BCL2, BCL2L1, BCL6, CASP10, CASP7, CEBPA, CSF3, CSF3R, CX3CR1, CXCL2, EGF, ERBB2, ERBB3, ERBB4, FAS, FASN, FLT1, FLT3, FOS, GNAS, IGF1, IGF1R, IL1A, IL1B, IL20RA, IL4, IL6, INPP5K, INSR, IRS1, JUN, KDR, KIT, KITLG, LIF, MAP3K8, MAPKAPK5, MAPT, MCL1, MDM4, MPL, MYB, NRG1, NTRK1, PDGFA, PDGFRA, PDPK1, PREX1, PRKD1, PRLR, RASGRP3, TEK, TGFB3, THPO, TNFRSF14, TNFSF10 |
| Increased  (initial_value = 1) | ACKR3, BAMBI, BIRC5, BNIP3, BRAF, CCL13, CCL5, CCND1, CCNG2, CCR2, CCR7, CD19, CD27, CD70, CD86, CDC25B, CDC42, CDH1, CDK2, CDK4, CDKN2A, CDKN2B, CEBPB, CFLAR, CHEK1, CREB3L2, CSF1R, CTNND1, CX3CL1, CXCL10, CXCL11, CXCL16, CXCL8, CXCL9, DDIT3, DDIT4, EGFR, EIF4EBP1, ETS1, GAPDH, GRB2, GSK3B, GTSE1, HIF1A, HSPB1, IGFBP3, INHBA, IRAK1, IRF3, LAMTOR3, LTBP1, LYN, MAP2K1, MAP2K2, MAP3K13, MAX, MET, MLLT4, MRAS, MYC, NFKB1, NRAS, PDGFB, PDGFC, PDGFRB, PDK1, PGK1, PIK3CA, PIM1, PLA2G4A, PLK1, PMAIP1, PRKX, PTGS2, PTK2, PTPN11, PTPRF, RAF1, RALBP1, RASGRP2, RASSF1, RASSF5, RHEB, RHOA, RPS6, RRAS, SERPINB5, SERPINE1, SIAH1, SLC2A1, SMO, SPI1, SPP1, SRC, STAT1, STMN1, SYK, TAB1, TAB2, TCF7, TFRC, TGFB1, TGFBR1, TGFBR2, THBS1, TIMP1, TNF, TNFRSF1A, TNFRSF1B, TRAF2, VEGF |
