# Supplementary material for: A Network-Based Data Integration Approach to Support Drug Repurposing and Multi-Target Therapies in Triple Negative Breast Cancer
Source: PLoS One. 2016 Sep 15;11(9):e0162407. doi: 10.1371/journal.pone.0162407 (PMC5025072; doi:10.1371/journal.pone.0162407)
Supplement: S3 Table — I: Imatinib, I+V: Imatinib combined with Vemurafenib, I+V: Imatinib combined with Flucytosine and I+V+F: Imatinib, Vemurafenib and Flucytosine combined all together. Note that no TP can occur when the drug used in combination with Imatinib does not have any target in the pathway. In the last rows the DrugEFF and the EFFECT indices are provided. (DOCX) [file pone.0162407.s003.docx]

S3 Table. PathEFF_MC_ index of each combination in every pathway.

| **Pathway** | **I** | **no I** | **I+V** | **no I+V** | **I+F** | **no I+F** | **I+V+F** | **no I+V+F** |
| --- | --- | --- | --- | --- | --- | --- | --- | --- |
| Chemokine | 0,621 | 0,548 | 0,666 | 0,548 | 0,599 | 0,548 | 0,632 | 0,548 |
| Cytokine-cytokin | 0,568 | 0,449 | no TP | no TP | no TP | no TP | no TP | no TP |
| ErbB | 0,7 | 0,585 | 0,679 | 0,585 | 0,663 | 0,585 | 0,698 | 0,585 |
| FoxO | 0,551 | 0,557 | 0,569 | 0,557 | 0,542 | 0,557 | 0,604 | 0,557 |
| HIF-1 | 0,568 | 0,554 | 0,589 | 0,554 | 0,586 | 0,554 | 0,592 | 0,554 |
| Insulin | 0,569 | 0,503 | 0,551 | 0,503 | no TP | no TP | 0,558 | 0,503 |
| Jak-Stat | 0,703 | 0,611 | no TP | no TP | no TP | no TP | no TP | no TP |
| MAPK | 0,552 | 0,503 | 0,555 | 0,503 | 0,564 | 0,503 | 0,559 | 0,503 |
| mTOR | 0,604 | 0,686 | 0,582 | 0,686 | no TP | no TP | 0,596 | 0,686 |
| p53 | 0,533 | 0,532 | 0,504 | 0,532 | 0,526 | 0,532 | 0,491 | 0,532 |
| Pathways in cancer | 0,503 | 0,572 | 0,515 | 0,572 | 0,503 | 0,572 | 0,526 | 0,572 |
| PI3K-Akt | 0,454 | 0,545 | 0,483 | 0,545 | 0,511 | 0,545 | 0,578 | 0,545 |
| Rap1 | 0,531 | 0,631 | 0,557 | 0,631 | no TP | no TP | 0,598 | 0,631 |
| Ras | 0,642 | 0,532 | 0,661 | 0,532 | no TP | no TP | 0,667 | 0,532 |
| TGF-beta | 0,372 | 0,408 | no TP | no TP | no TP | no TP | no TP | no TP |
| TNF | 0,503 | 0,38 | 0,514 | 0,38 | no TP | no TP | 0,526 | 0,38 |
| Toll-Like | 0,611 | 0,505 | 0,597 | 0,505 | no TP | no TP | 0,574 | 0,505 |
| VEGF | 0,48 | 0,503 | 0,808 | 0,503 | no TP | no TP | 0,787 | 0,503 |
| **DrugEFF** | **0,559** | **0,534** | **0,589** | **0,542** | **0,538** | **0,541** | **0,591** | **0,534** |
| **EFFECT** | **4,812** | | **8,534** | | **-0,573** | | **10,807** | |

The index was computed to assess if an increased effect occurs when Imatinib is combined with other drugs. I: Imatinib, I+V: Imatinib combined with Vemurafenib, I+V: Imatinib combined with Flucytosine and I+V+F: Imatinib, Vemurafenib and Flucytosine combined all together. Note that no TP can occur when the drug used in combination with Imatinib does not have any target in the pathway. In the last rows the DrugEFF and the EFFECT indices are provided.
