# Supplementary material for: A Network-Based Data Integration Approach to Support Drug Repurposing and Multi-Target Therapies in Triple Negative Breast Cancer
Source: PLoS One. 2016 Sep 15;11(9):e0162407. doi: 10.1371/journal.pone.0162407 (PMC5025072; doi:10.1371/journal.pone.0162407)
Supplement: S4 Table — (DOCX) [file pone.0162407.s004.docx]

S4 Table. Borda score to rank DP genes that can be elected to be monitored in in vitro experiments.

| Genes | Score |
| --- | --- |
| ABL1, ACACA, ADCY9, AKT1, AKT2, AREG, ARRB1, ATM, BAD, BAMBI, BCL2, BCL2L1, BCL6, BNIP3, BRAF, CASP10, CASP7, CBLB, CCL5, CCNB1, CCNB2, CCND1, CCND2, CCND3, CCNG2, CD19, CD86, CDC25B, CDC42, CDH1, CDK2, CDKN1A, CDKN2B, CEBPB, CFLAR, COL6A3, CREB3L2, CREBBP, CTNND1, CXCL10, CXCL11, CXCL8, CXCL9, DDIT3, DDIT4, EGF, EGFR, EGLN3, EIF4E, EIF4EBP1, ENO1, ERBB3, ERBB4, ETS1, ETS2, FAS, FASN, FBP1, FBXO25, FLNA, FLNB, FLOT1, FOS, G6PC3, GADD45A, GAPDH, GNAI1, GNAS, GRB2, GSK3A, GSK3B, HIF1A, HOMER3, HRAS, HSPB1, IFNAR1, IFNAR2, IFNGR1, IGF1, IGF1R, IL1A, IL1B, IL6, INPP5K, INSR, IRAK1, IRF3, IRS1, IRS4, JAK1, JUN, KDR, KRAS, LAMTOR3, LTBP1, MAGI1, MAP2K1, MAP2K2, MAP3K13, MAP3K2, MAP3K8, MAPK1, MAPK10, MAPK3, MAPK9, MAPKAPK5, MAPT, MAX, MCL1, MKNK2, MLLT4, MRAS, MYB, MYC, NCK1, NCK2, NFKB1, NRAS, NRG1, NTRK1, NTRK2, PDGFA, PDGFB, PDGFRA, PDGFRB, PDK1, PDPK1, PGK1, PIK3CA, PIM1, PKN1, PKN2, PLA2G4A, PLCG2, PLD1, PLK1, PREX1, PRKCA, PRKCB, PRKCD, PRKCI, PRKCZ, PRKD1, PRKX, PTEN, PTGS2, PTK2, PTPN11, PTPRF, PYGB, RAF1, RALB, RALBP1, RAP1A, RASGRP2, RASGRP3, RASSF1, RASSF5, RHEB, RHOA, RPS6, RPS6KB1, RRAS, SERPINE1, SLC2A1, SMAD7, SPP1, SPRED2, SPRY1, SRC, STAT1, STMN1, SYK, TAB1, TAB2, TFRC, TGFB1, TGFB3, TGFBR1, TGFBR2, THBS1, THPO, TIMP1, TNF, TNFRSF1A, TNFRSF1B, TNFSF10, TP53, TRAF2, ULK2, VAV3, VEGFA, ZFYVE16 | 1.00 |
| LYN | 0.75 |
| ERBB | 0.50 |
| ENO2, FLT1, PDHA1, TEK | 0.25 |
| EGLN2 | 0.00 |
