# Supplementary material for: A Network-Based Data Integration Approach to Support Drug Repurposing and Multi-Target Therapies in Triple Negative Breast Cancer
Source: PLoS One. 2016 Sep 15;11(9):e0162407. doi: 10.1371/journal.pone.0162407 (PMC5025072; doi:10.1371/journal.pone.0162407)
Supplement: S5 Table — (DOCX) [file pone.0162407.s005.docx]

S5 Table. Results of cell viability performed by treating MCF7 and MDA-MB-231 cell lines with different doses of Imatinib.

|  |  | **MCF7** | | |
| --- | --- | --- | --- | --- |
|  | day 0 | day 2 | day 5 | day 7 |
| control | 1 | 2,4232 | 2,6787 | 3,07545 |
| 5uM | 1 | 2,14485 | 2,7379 | 2,55535 |
| 10uM | 1 | 1,80385 | 2,58415 | 2,65315 |
| 15uM | 1 | 1,75055 | 2,58025 | 2,6949 |
|  | | | | |
|  |  | **MDA-MB-231** | | |
|  | day 0 | day 2 | day 5 | day 7 |
| control | 1 | 2,2218 | 3,05775 | 3,2978 |
| 5uM | 1 | 2,0044 | 3,1166 | 3,4455 |
| 10uM | 1 | 1,6994 | 2,7903 | 1,3425 |
| 15uM | 1 | 2,379 | 2,8157 | 0,9328 |
