# Supplementary material for: A Network-Based Data Integration Approach to Support Drug Repurposing and Multi-Target Therapies in Triple Negative Breast Cancer
Source: PLoS One. 2016 Sep 15;11(9):e0162407. doi: 10.1371/journal.pone.0162407 (PMC5025072; doi:10.1371/journal.pone.0162407)
Supplement: S1 Appendix — (DOCX) [file pone.0162407.s006.docx]

S1 Appendix.

TSDS score.

The Topological Score of Drug Synergy (TSDS) is a function that ranks target combinations based on the target topological features [1]. The TSDS is computed following 3 main steps:

1. *DP reachability*. Frist, the function ${TP}_{to}DP$ is calculated as in Eq. 1 in order to evaluate how a target $t$ reaches a Disease Protein $dp$.

${TP}_{to}DP(t, dp)=\frac{\sum_{sh=1}^{N_{sh}} {\prod_{i,j} w}_{ij}}{N_{sh}}$  (1)

$N_{sh}$is the total number of shortest paths between the target $t$and the Disease Node $dp$, $(i,j)$ is a pair of nodes that belongs to the shortest path $sh$ and $w_{ij}$is the weight on the edge between the two nodes. The function takes into account the weights on the shortest path’s edges. In this way, the nodes with the highest confidences in their predicted relationships.

1. *Global effect*. The function ${TP}_{to}allDP$ is then computed following Eq. 2.

${TP}_{to}allDP(t)=\sum_{d=1}^{N_{d}} \frac{{TP}_{to}DP}{N_{sh}}$  (2)

$N_{d}$is the total number of DP and ${TP}_{to}DP\left( t, dp \right)$ is calculated as in Eq. 1. Since ${TP}_{to}allDP$ quantifies the potential effect of a drug interacting with a target t on all the DP in the network, it allows to construct a ranked list of the targets taking into account their global effect on all the DP proteins.

1. *Synergistic effect*. Finally, the TSDS score is computed to obtain an ordered list of target combinations. The TSDS aims at simulating simultaneous actions of multiple-agents which potentially could act on different targets. For computational and therapeutic compliance reasons, the multi-target approach has been restricted to triplets of targets.

The proposed TSDS score is given by:

$TSDS(A,B,C)=\prod_{t\in(A,B,C)} {TP}_{to}allDP(t)$ (3)

where $(A,B,C)$ is a triplet of targets and ${TP}_{to}allDP(t$) is obtained using the Eq. 2. The score is calculated for all the possible combinations of 3 TPs in the network.

The resulting TDSD were normalized using the function:

$TSDS\_norm(A,B,C)=\frac{TSDS\left( A,B,C \right)-\max\left( TSDS \right)}{\max\left( TSDS \right)- min(TSDS)}$ (4)

where $\max\left( TSDS \right)$ and $\max\left( TSDS \right)$are the minimum and maximum values of the previously calculated TSDS, respectively.

**Selection of significant combinations.**

The selection of the most significant TSDS, leading to the identification of the best target combinations for a multi-target approach, is performed by constructing a null distribution of TSDS values. In order to validate the robustness of our constraints, the null distribution is built by computing the TSDS for 50,000 combinations of 3 proteins randomly selected from the complete set of nodes. A measure of the significance of the results is finally assessed by evaluating the null distribution area to the right of each TSDS observed. The combinations are selected as significant when they have a p-value < 0.01 [1].

1. Vitali F, Mulas F, Marini P, Bellazzi R. Network-based target ranking for polypharmacological therapies. J Biomed Inform. 2013;46: 876–881.
