# Supplementary material for: A Network-Based Data Integration Approach to Support Drug Repurposing and Multi-Target Therapies in Triple Negative Breast Cancer
Source: PLoS One. 2016 Sep 15;11(9):e0162407. doi: 10.1371/journal.pone.0162407 (PMC5025072; doi:10.1371/journal.pone.0162407)
Supplement: S2 Appendix — (DOCX) [file pone.0162407.s007.docx]

S2 Appendix.

Data Fusion.

Data fusion by matrix tri-factorization (DFMF) is an intermediate data integration approach meaning that it does not merge the input data, nor it builds separate models for each data sources [1]. In fact, it addresses the multiplicity of data and fuses them through inference into a single joint model. Currently, this particular approach is often preferred in many applications because of its superior predictive accuracy [1–4].

This approach, which extends a strategy used in recommender systems, considers any data set or data source as a graph and, consequently, it can be represented through its adjacency matrix. Such data matrices are then simultaneously factorized to reveal hidden associations.

The DFMF consists in three main steps:

1. *Construction of relation and constraint matrices from all available data.*

The DFMF considers *r* object types $\xi_{1}\ldots\xi_{r}$ and a collection of data sources, each relating a pair of object types ($\xi_{i},\xi_{j}$). In a real-world scenario we can have associations between objects of different types as well as relations between objects of the same types. Data sources corresponding to associations between objects of different types (i.e. ($\xi_{i},\xi_{j}$) where $\neq j$ ) are represented through $R_{i,j}$matrices that constitute a block of a complete relationship matrix $R$. An example of such a matrix would relate drug-target interactions. On the other hand, data sources of associations relating objects of the same type $\xi_{i}$are represented by a constraint matrix $\theta_{i}$. Constraints are collectively encoded in a set constraint block diagonal matrix $\theta$. Examples of such constraints are protein-protein interactions. The $R$ and $\theta$ matrices are used as inputs of the data fusion:

$R= \left[ \begin{aligned} \begin{matrix} 0 & R_{1,2} & \begin{matrix} \ldots& R_{1,r} \end{matrix} \\ R_{2,1} & 0 & \begin{matrix} \ldots& R_{2,r} \end{matrix} \\ \vdots& \vdots& \begin{matrix} \ddots& \vdots\end{matrix} \end{matrix} \\ \begin{matrix} R_{r,1} & R_{r,2} & \begin{matrix} \ldots& 0 \end{matrix} \end{matrix} \end{aligned} \right] \theta= \left[ \begin{aligned} \begin{matrix} \theta_{1} & 0 & \begin{matrix} \ldots& 0 \end{matrix} \\ 0 & \theta_{2} & \begin{matrix} \ldots& 0 \end{matrix} \\ \vdots& \vdots& \begin{matrix} \ddots& \vdots\end{matrix} \end{matrix} \\ \begin{matrix} 0 & 0 & \begin{matrix} \ldots& \theta_{r} \end{matrix} \end{matrix} \end{aligned} \right]$  (1)

1. *Simultaneous factorization of the relation matrices under given constraints.*

The block matrix $R$ is tri-factorized into block matrix factor $G$and $S$:

$G= \left[ \begin{aligned} \begin{matrix} G_{1}^{n_{1}\times k_{1}} & 0 & \begin{matrix} \ldots& 0 \end{matrix} \\ 0 & G_{2}^{n_{2}\times k_{2}} & \begin{matrix} \ldots& 0 \end{matrix} \\ \vdots& \vdots& \begin{matrix} \ddots& \vdots\end{matrix} \end{matrix} \\ \begin{matrix} 0 & 0 & \begin{matrix} \ldots& G_{2}^{n_{r}\times k_{r}} \end{matrix} \end{matrix} \end{aligned} \right] S= \left[ \begin{aligned} \begin{matrix} 0 & S_{1,2}^{k_{1}\times k_{2}} & \begin{matrix} \ldots& S_{1,r}^{k_{1}\times k_{r}} \end{matrix} \\ S_{2,1}^{k_{2}\times k_{1}} & 0 & \begin{matrix} \ldots& S_{2,r}^{k_{2}\times k_{r}} \end{matrix} \\ \vdots& \vdots& \begin{matrix} \ddots& \vdots\end{matrix} \end{matrix} \\ \begin{matrix} S_{r,1}^{k_{r}\times k_{1}} & S_{r,2}^{k_{r}\times k_{2}} & \begin{matrix} \ldots& 0 \end{matrix} \end{matrix} \end{aligned} \right]$ (2)

Such matrix factors are characterized by low dimensions with respect to the original R matrix and correspond to “latent” dimensions, i.e. meta-genes, meta-proteins. The factors are found by means of suitable optimization algorithms and thus by taking into account the whole relations structure. A factorization rank $k_{i}$ is assigned to each $i$ during inference of the factorized system. The estimation of such factors can be performed based on the available background knowledge (e.g. how many groups/latent dimensions are expected) and or with suitable heuristics. Among the latter, there is no clear consensus about the best strategy. Examples include the use of the cophenetic index or of principal component analysis.

$S_{i,j}$factors define the relations between two objects, while $G_{i}$ are specific for each objects type. These factors are then used to reconstruct a new $\hat{R}_{i,j}= G_{i}S_{i,j}G_{j}^{T}$considered an approximation of the original $R_{i,j}.$

R decomposition is obtained through a convergent iterative process, designed to minimize the following formula:

$J= \sum_{i,j} \left\| R_{i,j}-\hat{R}_{i,j} \right\|^{2}+tr(G^{T}\theta G)$ (3)

where $i$ and $j$ are $R$ blocks indices; $\hat{R}$ is the iteratively approximated R, $\left\| \cdot\right\|$ and $tr(\cdot)$ are the Frobenius norm and the trace, respectively. Because of random initialization, the whole process has to be repeated several times, in order to obtain a consensus matrix as a result.

1. *Identifications of predicted associations.*

Once the iterative process has concluded, the relation matrix $\hat{R}_{i,j}$for a target pairs of object types$i$ and $j$ is then taken into account to analyze novel and predicted associations between its elements. Each object pair $(o_{i},o_{j})$ represented in $\hat{R}_{i,j}$ is analyzed in order to identify pairs for which the predicted degree of relation is unusually high.

To this end, DFMF considers $(o_{i},o_{j})$ pairs whose association score in the new $\hat{R}_{i,j}$ is greater than the mean score of all known relations in the original $R_{i,j}$. Formally:

$\hat{R}_{i,j}\left( p,q \right)> \frac{1}{\left| A(o_{p}^{i},\xi_{j}) \right|}\sum_{o_{m}^{j}\epsilon A(o_{p}^{i},\xi_{j})} \hat{R}_{i,j}\left( p,m \right)$ (4)

where $A\left( o_{p}^{i},\xi_{j} \right)$is the set of all objects of $\xi_{j}$ related to $o_{p}^{i}$. This rule is row-centric, that is, given an object of type $\xi_{i}$, it searches for objects of the other type $\xi_{j}$ that it could be related to. Such rule can be modified to become column-centric or it is even possible to combine the two rules.

1. Zitnik M, Zupan B. Data Fusion by Matrix Factorization. IEEE Trans Pattern Anal Mach Intell. 2015;36: 41–53. Available: http://www.researchgate.net/publication/273647495_Data_Fusion_by_Matrix_Factorization

2. Žitnik M, Janjić V, Larminie C, Zupan B, Pržulj N. Discovering disease-disease associations by fusing systems-level molecular data. Sci Rep. Nature Publishing Group; 2013;3: 3202. doi:10.1038/srep03202

3. Wang F, Li T, Zhang C. Semi-Supervised Clustering via Matrix Factorization. SDM. 2008; Available: http://epubs.siam.org/doi/abs/10.1137/1.9781611972788.1

4. Li T, Zhang Y, Sindhwani V. A non-negative matrix tri-factorization approach to sentiment classification with lexical prior knowledge. Proc Jt Conf …. 2009; Available: http://dl.acm.org/citation.cfm?id=1687914
